# Emotional Intelligence, Self-Regulation, Smartphone Addiction: Which Relationship With Student Well-Being and Quality of Life?

**DOI:** 10.3389/fpsyg.2020.00375

**Published:** 2020-03-06

**Authors:** Maria Lidia Mascia, Mirian Agus, Maria Pietronilla Penna

**Affiliations:** Department of Pedagogy, Psychology, Philosophy, Faculty of Humanities, University of Cagliari, Cagliari, Italy

**Keywords:** adolescence, smartphone addiction, self-regulation, emotional intelligence, well-being, quality of life

## Abstract

This study emphasizes the importance of analyzing factors that contribute to student well-being, as a result of the multiplicity of factors that can affect their quality of life (QoL). The literature indicates that, among these factors, emotional intelligence and self-regulation play a central role in influencing adolescents’ psychological and scholastic well-being. Technology is a fundamental aspect of adolescent life but addiction to the use of smartphones is increasing, which can affect both emotional intelligence and self-regulation, and in turn individual well-being and QoL. Therefore, this study explores the role of smartphone use with respect to these aspects. Participants were 215 Italian students attending middle school. By applying partial least squares structural equation modeling (PLS-SEM), the results confirm that self-regulation affects the QoL of students, but its role varies according to the degree of smartphone addiction. In conclusion, we confirm the relevance of the relationship between self-regulation and smartphone addiction in teaching students to be aware of their time spent using smartphones. Emotional intelligence and, in general, self-regulation should be encouraged to support the well-being and QoL of students in their adolescence at school.

## Introduction

The well-being of students at school is a primary concern for teachers and educators (Stefansson et al., 2018) as it is strictly related to their quality of life (QoL: Camfield and Skevington, 2008). Several studies (Shoshani et al., 2016; Navarro et al., 2017) examine factors that can positively influence student well-being and QoL in adolescence, finding it to be the result of a combination of affective, behavioral and cognitive dimensions. Some literature shows a link between emotional intelligence and well-being (Zeidner and Olnick-Shemesh, 2010), particularly at class level (Balluerka et al., 2016). Another fundamental dimension connected to these two aspects is self-regulation (Thomas et al., 2019). Self-regulation strategies facilitate students’ planning and goal-setting prior to learning by enhancing their attention-focusing and self-monitoring processes (self-reflection) during learning or task performance (Zimmerman, 2002; Cleary and Chen, 2009).

Digital society provides numerous opportunities but despite the implied advantages it also brings risks, especially for younger people (Machimbarrena et al., 2019); indeed, use of the internet can become problematic, leading to consequences for personal well-being. In particular, young people are continually increasing their smartphone use (Humphreys et al., 2013) and internet addiction has become ubiquitous (Haverlag, 2013; Yam et al., 2019). A body of research states that problematic internet use can become addictive but the issue of smartphone use is more complex; undeniably, smartphones can link to the internet and also execute various types of applications (e.g., gaming, gambling, social media use, etc.), consequently causing psychological impairment (Lin et al., 2019; Yam et al., 2019). Adolescents between 16 and 18 years old were less likely to believe in the negative impact of the internet on health than older people (Do et al., 2020). The prevalence of internet addiction is 1.2–4.9% (Mak et al., 2014) in adolescents and as high as 30% in university students (Zhang and Ho, 2017). Most studies on this issue focus on describing behaviors and consequences, including depression, anxiety, alcohol misuse, musculoskeletal discomfort, and sleep problems (Bianchi and Phillips, 2005; Ho et al., 2014; Yang et al., 2017; Zhang and Ho, 2017; Alimoradi et al., 2019; Chen et al., 2020). Generally, studies emphasize that internet addiction is inversely related to the global Life Satisfaction Index (Cheng et al., 2018) and health-related QoL (Tran et al., 2017), leading to the need to spend increasing time on internet gaming and losing interest in hobbies, relationships, and educational opportunities (Ho et al., 2014). Many studies emphasize that self-regulation constructs are adversely affected by smartphone addiction (van Deursen et al., 2015), but self-regulation may contribute to the suppression of addictive behavior (Baumeister and Vonasch, 2015). Other studies hypothesize that people who are able to express and understand emotions and regulate feelings are better adjusted psychologically and socially and have a high level of well-being (Gascó et al., 2018), therefore it is important to preserve this dimension.

**FIGURE 1:**
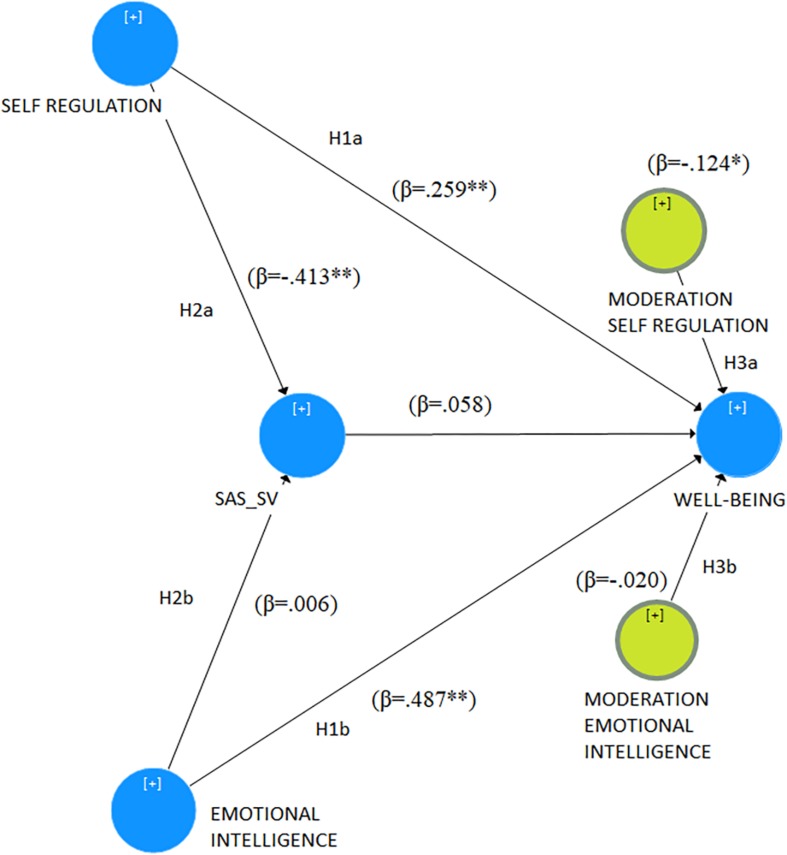
Conceptual framework: Results from PLS-SEM. H1a, hypothesis 1a; H1b, hypothesis 1b; H2a, hypothesis 2a; H2b, hypothesis 2b; H3a, hypothesis 3a; H3b, hypothesis 3b; SAS-SV, Smartphone Addiction Scale-Short version; β, Beta coefficient; ^∗^*p* < 0.05; ^∗∗^*p* < 0.01.

What is the relationship between these variables? These premises underline the need for attention to factors that can positively or negatively affect adolescent well-being. This study considers the effects of self-regulation (hypothesis 1a, H1a) and emotional intelligence (H1b) on scholastic well-being. The innovation in this model relates to the role that smartphone dependence plays in these relationships. We assess if smartphone dependence might mediate the effects of self-regulation (H2a) and emotional intelligence (H2b) on scholastic well-being. Furthermore, it is of interest to evaluate the potential moderating effect of smartphone addiction on the relation between self-regulation and well-being (H3a) and between emotional intelligence and well-being (H3b) (Figure 1). These hypotheses are based on previous research findings in the literature (Zimmermann and Iwanski, 2014; Verzeletti et al., 2016; Chung, 2019; Xu et al., 2019).

## Method

### Participants

This study involved 215 students (mean age 12.7 years; SD = 0.90) attending their last year of middle school in Sardinia (Italy).

### Measures and Procedure

The survey was conducted in the third-year classes of state middle schools during the school timetable, subject to agreement from parents, the headmaster and teachers. Informed consent was given by the students’ parents after the features and aims of the study had been explained to them. We established an atmosphere of participation and trust in all classes, allowing the students to choose to participate in the research and motivating them sufficiently for the purpose of the study. To prevent teachers from interfering during the survey, they were asked to adopt a neutral stance if they were present in the classroom. All teachers were helpful and cooperative, leaving the management of the survey to the provider. Data collection was carried out during school hours from 8:30 to 13:00. Administration of the questionnaire took 25–45 min, preceded by a detailed explanation of the objectives of the survey, the structure of the questionnaire, the method of compilation and the anonymity of the test. The study was approved by the ethics committee at the University of Cagliari, Italy (the Department of Pedagogy, Psychology, Philosophy).

The study protocol comprises five sections:

(1) A questionnaire on socio-demographic characteristics collects specific information such as age and educational level.
(2) The *self-regulation* questionnaire (Moè and De Beni, 2000) aims to identify the components of the self-regulation approach to the study, with particular reference to three meta-cognitive dimensions: processing skills, organization and self-evaluation. Research shows how students can organize their study activities with a time-bound work programe that complies with commitments and deadlines (Ley and Young, 1998; Moè and De Beni, 2000), using schema-driven strategies (based on schematization, building diagrams, and tables, notepads, etc.) and the adoption of specific processing methods. Successful students are aware of their own study method, know how to properly assess their own preparation and are more likely to reflect on the best way to deal with their studies. The scale consists of 30 items (10 items for each dimension) to be answered on a Likert scale from 1 to 5, with higher values denoting better skills. Cronbach’s alpha coefficient for reliability is good for all dimensions: processing skills, α = 0.81; organization, α = 0.76; self-evaluation, α = 0.72.
(3) The “My Life as a Student” questionnaire (Soresi and Nota, 2003; Nota et al., 2011) allows students to explore their levels of satisfaction and *well-being*. This instrument consists of 26 items (on a five-point Likert scale, with higher scores indicating greater well-being) and examines seven satisfaction factors in several aspects: the school experience (α = 0.86); opportunities to make autonomous decisions (α = 0.66); relationships with classmates (α = 0.70); current living conditions (α = 0.76); family relationships (α = 0.71); praise received when due (α = 0.72); and availability of assistance (α = 0.79).
(4) The Emotional Intelligence Scale (EIS) questionnaire (Schutte et al., 1998), conducted in a validated Italian version (Ciucci et al., 2009), is designed to determine *emotional intelligence* and consists of 33 closed-ended statements (five-point Likert scale, with higher values representing improved emotional intelligence) such as “I am aware of my emotions as I experience them.” The three scales identified in the questionnaire measure: emotional appraisal and expression of oneself (13 items, α = 0.64), and others (10 items, α = 0.68); and regulation of emotions in oneself and others (10 items, α = 0.71).
(5) The Smartphone Addiction Scale (SAS) is a validated questionnaire designed to determine the risk level of *smartphone addiction* and identify high-risk groups among adolescents in Korea (Kwon et al., 2013). A short version (SAS-SV) was conducted, validated in Italy by De Pasquale et al. (2017). The questionnaire includes 10 questions (α = 0.79) describing daily disruptions in life, positive expectations, withdrawal, relationships in cyberspace, overuse and tolerance. Participants express their opinions on a six-point scale (1 = strongly disagree; 6 = strongly agree), with higher scores designating greater smartphone addiction (De Pasquale et al., 2017).

In order to verify the research hypotheses, this work proposed a conceptual model including those dimensions that might potentially affect student well-being. The model was devised on the basis of previous relevant works in the literature (Zimmermann and Iwanski, 2014; Gascó et al., 2018; Machimbarrena et al., 2019; Do et al., 2020). The conceptual model was assessed by applying component-based partial least squares structural equation modeling (PLS-SEM), designed to determine the values of the variables in relation to the predicted purpose (Chin, 1998). In this work PLS-SEM was used as the main statistical technique to evaluate our model due to the multiplicity of constructs and relationships to be assessed (Hair et al., 2011).

This statistical approach is particularly suitable for small samples, demonstrates robustness of non-normal data and has fewer restrictive assumptions than factor-based SEM. PLS-SEM analyses both the outer measurement model (referring to the quality, reliability and validity of the construct under study) and the inner model (where paths between latent variables are estimated) (Hair et al., 2012; Sarstedt et al., 2017). Statistical analyses are performed using the software R 3.6.1 (R Core Team, 2019) and Smart-PLS (V.3.2.8) (Ringle et al., 2015). In the model assessed, the subscales related to the constructs of *self-regulation* (processing skills; organization; self-evaluation) (Moè and De Beni, 2000) and *emotional intelligence* (emotions related to others and themselves; regulation and use of emotions) (Ciucci et al., 2009) were used as observed variables. For *smartphone addiction* on the SAS-SV (De Pasquale et al., 2017), all 10 items that explore the construct were used as observed variables. *Well-being* was measured by the seven subscales identified on the “My Life as a Student” questionnaire (Soresi and Nota, 2003).

## Results

Descriptive statistics were performed on each variable to evaluate the distribution (Table 1). PLS-SEM was then performed with a reflective measurement model (Hair et al., 2019). Table 2 illustrates the indicators used for the outer measurement model. The factor loadings obtained vary from to 0 458 to 0.862 for all constructs; the consistent reliability coefficient Rho_A was consistently greater than 0.7, which indicates an acceptable internal reliability for the dimensions (Dijkstra and Henseler, 2015). The constructs indicated an average variance extracted (AVE) value higher than 0.5, indicating convergent validity (Fornell and Larcker, 1981). The adjusted *R*^2^ value was 0.161 for *smartphone addiction* and 0.390 for *well-being*, highlighting weak and moderate effects, respectively (Hair et al., 2011).

**TABLE 1:** Descriptive statistics for the queried variables.

	Variables	Category			Fr (%)		

	Gender	Female			106 (49.3%)		

		Minimum	Maximum	Mean	Standard deviation	Skewness	Kurtosis
	Age	10	15	12.7	0.907	–0.172	0.564
Self-regulation	Processing skills	1.60	5.00	3.29	0.736	0.011	–0.466
	Organization skills	2.20	4.80	3.53	0.520	0.001	–0.254
	Self-evaluation skills	2.30	4.30	3.27	0.429	–0.030	–0.412
Emotional intelligence	Appraisal and expression of emotion in the self	2.45	4.91	3.59	0.537	0.102	–0.420
	Appraisal and expression of emotion in the others	1.50	5.00	3.68	0.786	–0.190	–0.541
	Regulation and use of emotions	1.44	5.00	3.81	0.639	–0.405	0.020
Smartphone addiction	Smartphone addiction	10.00	44.00	22.90	8.80	0.381	–0.757
Scholastic well-being	Satisfaction with the School experience	7.00	35.00	26.80	6.02	–0.521	–0.327
	Satisfaction with opportunities to make decisions autonomously	5.00	25.00	17.10	3.84	–0.309	0.255
	Satisfaction with relationships with classmates	3.00	15.00	11.00	2.81	–0.668	0.080
	Satisfaction with Current life conditions	3.00	15.00	8.38	2.89	0.164	–0.478
	Satisfaction with relationships with family members	4.00	20.00	7.80	3.37	1.220	1.540
	Satisfaction with praise received when due	2.00	10.00	5.86	1.88	0.072	–0.499
	Satisfaction with help availability	2.00	10.00	3.96	1.97	1.010	0.631

*Fr, frequency.*

**TABLE 2:** PLS-SEM: Outer model.

Construct	Observed variables	Latent variable loadings	Rho_A	Average variance extracted	Adjusted *R*^2^
Self-Regulation	Processing	0.699	0.700	0.578	
	Organization	0.708			
	Self-evaluation	0.862			
Emotional Intelligence	Appraisal and expression of emotion in the self	0.711	0.707	0.621	
	Appraisal and expression of emotion in the others	0.803			
	Regulation and use of emotions	0.844			
Dependence on smartphone	Sas item1	0.652	0.881	0.457	0.161
	Sas item2	0.520			
	Sas item3	0.458			
	Sas item4	0.664			
	Sas item5	0.791			
	Sas item6	0.700			
	Sas item7	0.757			
	Sas item8	0.691			
	Sas item9	0.786			
	Sas item10	0.666			
Well-being	School experience	0.761	0.836	0.475	0.390
	Opportunities to make decisions autonomously	0.695			
	Relationships with classmates	0.708			
	Current life conditions	0.502			
	Relationships with family members	792			
	Praise received when due	0.589			
	Help availability	0.732			

*Rho_A, consistent reliability coefficient; SAS, smartphone addiction scale.*

Concerning the inner model, each path is calculated and assessed by applying the bootstrapping routine (5000 subsamples from the original data), calculating standard errors, *T* values and *p* values. This procedure identifies the significance of each relationship and effect (Hair et al., 2019; Table 3). Specifically, the positive effects of *self-regulation* (H1a) (β = 0.259^∗∗^) and *emotional intelligence* (H1b) (β = 0.487^∗∗^) on *well-being* are confirmed (Table 3). Although the negative effect of *self-regulation* on *smartphone addiction* is confirmed (β = −0.413^∗∗^), the influence of *smartphone addiction* on *well-being* has not been established as an indirect overall effect, which does not support H2a. H2b has not been confirmed, highlighting that there is no indirect overall effect between *emotional intelligence, smartphone addiction*, and *well-being*. Furthermore, the findings emphasize a significant negative moderation effect of *smartphone addiction* on the relationship between *self-regulation* and *well-being* (H3a) (β = −0.124^∗^). The moderation effect of *smartphone addiction* on the relationship between *emotional intelligence* and *well-being* (H3b) has not been confirmed (Table 3).

**TABLE 3:** PLS-SEM: Inner model.

Hypothesis	Relationship	Standardized beta	Mean	Standard deviation	*T*-value	*p*	Decision
H1a	Self-regulation ->Well-being	0.259	0.257	0.086	3.014	0.003	Supported
H1b	Emotional intelligence ->Well-being	0.487	0.476	0.131	3.731	<0.0001	Supported
H2a	Self-regulation ->Dependence on smartphone	–0.413	–0.423	0.056	7.405	<0.0001	supported
	Dependence on smartphone ->Well-being	0.058	0.049	0.062	0.933	0.351	Not supported
	Total indirect effect Self-regulation ->Dependence on smartphone ->Well-being	–0.024	–0.021	0.026	0.900	0.368	Not supported
H2b	Emotional intelligence ->Dependence on smartphone	0.006	0.005	0.083	0.070	0.944	Not Supported
	Total indirect effect Emotional-intelligence ->Dependence on smartphone ->Well-being	0.000	0.002	0.007	0.049	0.961	Not supported
H3a	Moderation Dependence on smartphone on Self-regulation ->Well-being	–0.124	–0.117	0.062	2.018	0.044	Supported
H3b	Moderation Dependence on smartphone on Emotional intelligence ->Well-being	–0.020	–0.015	0.060	0.335	0.738	Not supported

*H1a, hypothesis 1a; H1b, hypothesis 1b; H2a, hypothesis 2a; H2b, hypothesis 2b; H3a, hypothesis 3a, H3b: hypothesis 3b; p, probability.*

## Discussion and Conclusion

The findings of this work highlight the multivariate relationships affecting adolescent well-being, including the role played by their dependence on smartphones. To the best of our knowledge, few works in the literature have referred to the relation between smartphone addiction, emotional intelligence, self-regulation and well-being. The literature features a series of studies showing that many factors influence well-being and QoL in adolescence (Jovanović, 2016). QoL and satisfaction are defined as cognitive components of subjective well-being (Diener et al., 1999). Many scholars emphasize the multiplicity of factors concerning the emotional and self-regulation processes of young adolescents (e.g., Abe, 2011). Furthermore, recently it has been highlighted that in our daily lives (work, school, leisure) attention is often directed to smartphones. There are many advantages to using technology but the excessive use of smartphones for continuous connectivity can lead to internet addiction (Tonioni and Corvino, 2011) and to the alarming phenomenon of hikikomori (Suwa and Suzuki, 2013).

These relevant facts support the necessity to deepen our knowledge of the relationship between smartphone addiction and well-being, specifically in adolescents. The current model assessed relationships that have seldom been tested empirically before (e.g., mediation and moderation effects of smartphone addiction in the relationships between self-regulation and well-being and between emotional intelligence and well-being). This study attempted to identify the dimensions affecting adolescent well-being and has highlighted some interesting insights. In a closer look at the relationships between the variables that underlie scholastic QoL, our findings confirm the positive effect of *self-regulation* and *emotional intelligence* on *well-being*. The negative effect of *self-regulation* on *smartphone addiction* was also highlighted. However, the indirect effects of *self-regulation*, *smartphone addiction*, and *well-being* have not been established. Moreover, the indirect overall effects of *emotional intelligence, smartphone addiction*, and *well-being* have not been confirmed. It is of interest that the results emphasize significant negative moderation effects of *smartphone addiction* on the relationship between *self-regulation* and *well-being*, highlighting that the effect of self-regulation on well-being can vary depending on the level of *smartphone addiction*. Specifically, this last significant moderation effect implies that a low level of smartphone addiction enhances the positive relation between self-regulation and well-being; on the other hand, when smartphone addiction is high, the positive relationship between self-regulation and well-being is weakened. These findings shed light on issues that should be taken into consideration to improve adolescent well-being.

Moreover, it should be pointed out that some limitations of these findings might derive from the cross-sectional research design, the non-probabilistic sampling method in the Italian context and the presentation of self-report questionnaires.

New technologies offer endless possibilities for students and schools, but we must find ways to benefit, depending on the level of smartphone addiction. For example, smartphone applications can be used to deliver immersive virtual reality therapy for treating internet addiction in adolescents (Zhang et al., 2017). Education must play an active role in helping digital natives learn about and use these new tools. Emphasis should be placed on education concerning emotional intelligence and self-regulation in order to achieve psychological and social well-being, and in turn global life satisfaction (Huebner et al., 2005).

## Data Availability Statement

The datasets for this study are available from corresponding author on reasonable request.

## Ethics Statement

The studies involving human participants were reviewed and approved by the Department of Pedagogy, Psychology, Philosophy, Faculty of Humanities, University of Cagliari, Cagliari, Italy. Written informed consent to participate in this study was provided by the participants’ legal guardian/next of kin.

## Author Contributions

MM and MP contributed to the design of the study. MA analyzed the data. All authors wrote, read, revised and approved the final manuscript.

## Conflict of Interest

The authors declare that the research was conducted in the absence of any commercial or financial relationships that could be construed as a potential conflict of interest.
